# The effectiveness of web-based training for parents on post-traumatic stress disorder in children

**DOI:** 10.3389/fpsyg.2024.1325475

**Published:** 2024-03-08

**Authors:** Zakieh Omidvar Eshkalak, Soroor Parvizy, Naima Seyedfatemi, Hamid Haghani, Hadis Nazari

**Affiliations:** ^1^School of Nursing and Midwifery, Iran University of Medical Sciences, Tehran, Iran; ^2^Department of Biostatistics, School of Nursing and Midwifery, Iran University of Medical Sciences, Tehran, Iran; ^3^Department of Pediatric Nursing, School of Nursing and Midwifery, Ahvaz Jundishapur University of Medical Sciences, Ahvaz, Iran

**Keywords:** stress disorders, post-traumatic, e-learning, parents, child, education, nurse

## Abstract

**Introduction:**

Post-traumatic stress disorder (PTSD) after an injury such as accidents is common in children and can affect their overall physical and mental functioning and quality of life. Early intervention can have significant health benefits for children. This study aimed to investigate the effectiveness of web-based training for parents on post-traumatic stress disorder in children.

**Method:**

This was a quasi-experimental study with intervention and control group. 110 parents of children aged 10–18 years with PTSD after a traumatic event were selected through available sampling and assigned to intervention and control groups. Data was collected by a researcher-made demographic questionnaire and the Child Revised Impact of Events Scale (CRIES-8). Parents in the intervention group received a 4-week training course through a researcher-designed website, but the control group received routine care by the clinical team, which the main focus of care and training was on the physical aspects of the disease, and no intervention was done for PTSD. Two weeks after the intervention, the level of child stress was measured and compared in both groups. Data were analyzed using SPSS V.22.

**Results:**

The difference between the mean score of total traumatic stress and its subscales before intervention was not statistically significant (*p* = 0.23). But after intervention, the mean score of total traumatic stress and its subscales decreased in the intervention group and increased in the control group and this difference was statistically significant (*p* < 0.001).

**Conclusion:**

E-learning parent training has the potential to support children with PTSD. This available and cost-effective procedure can be recommended to help children with PTSD and possibly increase recovery in these patients.

## 1 Introduction

### 1.1 Post-traumatic stress disorder in children

PTSD (Post-Traumatic Stress Disorder) is a disorder that occurs when a child is exposed to actual or threatened death, serious injury such as accidents or sexual violence (U. S. Department of Health and Human Services, 2016). Accidents are one of the most important causes of injury and hospital admission in children (Hockenberry et al., 2016), and PTSD is one of the most common mental disorders in children with injuries. Most children and their parents have reported at least one severe PTSD reaction during the first month after a traumatic event (Marsac et al., 2016; Zhang et al., 2018). A recent meta-analysis reported the prevalence of PTSD in children to be 15.9% (Alisic et al., 2014). Also in another study, 31.1% of children up to the age of 18 experienced trauma, of which 7.8% had undergone PTSD.

PTSD is diagnosed when a person who experienced an injury shows symptoms such as recurrent distressing dreams or memories, persistent avoidance of stimuli associated with the traumatic event (e.g., riding a bicycle or getting in a vehicle), and negative alterations in cognitions and mood associated with the traumatic event. These symptoms may last for up to 1 month, but if no action is taken, it becomes PTSD. The disturbance causes clinically significant distress or impairment in social, occupational, or other important areas of functioning (U. S. Department of Health and Human Services, 2016; Lewis et al., 2019). As studies reported PTSD is strongly associated with behavioral and emotional, interpersonal problems, drug abuse, academic failure, sleep disorders, depression, anxiety, ADHD (Attention Deficit Hyperactivity Disorder), and impaired quality of life (Kovachy et al., 2013; Alisic et al., 2014; Marsac et al., 2016; Salari et al., 2017; Lewis et al., 2019; Xiang et al., 2021). So, identification and treatment of PTSD children is of vital importance.

### 1.2 Existing treatment of PTSD in children

Treatment mainly includes psychotherapy (e.g., Cognitive Behavioral Therapy) and pharmacotherapy. Recently, more efforts have focused on secondary prevention of existing symptoms through early intervention. Early preventive interventions during the first few days of symptom onset both reduce and prevent acute PTSS (Post-Traumatic Stress Symptoms) and prevent the onset of chronic PTSD (Kassam-Adams et al., 2016).

The treatment of affected children is multifaceted and should include the interventions of both the child and parents (Kaminer et al., 2005). But it seems that focusing on parents may be more effective because parents are a major source of support for children after an injury (Marsac et al., 2013); and the extent of parent’s discomfort following a child trauma, affected children’s ability to adjust emotionally and control pain or trauma. Parental response to a traumatic event in a child plays an important role in the psychological recovery of the child after an accident (De Young et al., 2014). In addition, maladaptive family functioning is one of the causes of PTSD in children after experiencing a traumatic event (Dorrington et al., 2019). On the other hand, parents are extremely weak in identifying acute PTSS in their children (Marsac et al., 2013). Therefore, it seems that intervention for parents and increasing their awareness can help a child with PTSD; which is consistent with the family-centered care approach in child care. This intervention can be implemented in the form of a web-based psychological training program.

Rapid advances in internet access are not only paving the way for a dynamic mode of delivering psychological empowerment interventions but can also be widely disseminated and implemented (Grey et al., 2013). E-learning in addition to being always available, there is no need to visit in person and it is cost-effective.

Internet-based psychosocial education interventions have been effective for a wide range of populations with physical and psychiatric problems (Marsac et al., 2013; Fidika et al., 2015; Kenardy et al., 2015; Dam et al., 2019); but some studies have shown contradictory results that e-learning is not effective in some situations such as improving the level of mental health literacy of mothers of adolescents with psychosis (Nakanishi et al., 2023) Or to help mothers with children with food allergies (Sugunasingha et al., 2022), Also as far as we know, research into interventions for PTSD in children has only paid to behavioral therapy (Mahmoudi-Gharaei et al., 2007), play therapy (Shamsi pour et al., 2019) or desensitization (Moghadam et al., 2017) for the child, while parents, as the primary focus of child life, have been overlooked. Also, a few of these studies have provided Internet-based training.

### 1.3 The current study

The purpose of this study was to investigate the effects of parents’ web-based training on the level of post-traumatic stress symptoms in children. In this study, firstly, the focus of the intervention is on the parents of children with PTSD, secondly, the intervention is carried out as E-learning for parents, which has received a lot of attention today, and in addition, studies have reported different results about its effect; And thirdly, to better understand the effect of E-learning, a control group of parents that receives routine interventions was considered. We hypothesize that the parents’ web-based training is effective and the PTSS in the children of parents in the intervention group will be significantly reduced compared to the control group. If the hypothesis is confirmed, web-based training for parents can be used as an available and cost-effective method to help children with PTSD.

## 2 Materials and methods

### 2.1 Design

This is a quasi-experimental study with intervention and control group. The study was carried out at Shahid Rajaee Hospital, the main trauma center, in Qazvin, Iran.

### 2.2 Participants

The participants of the study consisted of all traumatized children and one of their parents (the father or mother who had more contact and provided much care for the child).

The participants’ inclusion criteria were as follows: age range of 10–18, hospitalization of a child due to a traumatic accident including crashes, car and motor vehicle accidents, limb fractures, multiple injuries, vein injuries, visceral injuries, burns and mild head injuries (GCS: Glasgow Coma Scale >14), child hospitalization 3 days to one-month post-traumatic injury, score above 17 on the Child Revised Impact of Events Scale (CRIES-8) questionnaire, and no evidence of parental abuse due to child injury. Parents’ inclusion criteria were: the ability to read and write in Persian, holding a high school diploma, the ability to use the internet, surf the web, and handle electronic devices, and the possession of no known mental or physical impairment. Exclusion criteria included: child death during the study, GCS decline, and change in child’s level of consciousness after entering the study and unwillingness to the continuation of the study.

### 2.3 Sample size and sampling

The sample size was determined at 95% confidence level with 80% test power and 102 participants (51 in each group). Finally, a total of 110 individuals were included in the study using the available sampling method. To prevent mixing of samples and data contamination, a 15-day interval between the selection of the control and intervention group was considered, as a result, 15 days after the discharge of the last sample of the control group, the intervention group was sampled.

### 2.4 Instruments

The instrument used in this study was a two-part questionnaire. The first part was a researcher-made demographic questionnaire that was developed based on the literature review and under the supervision of pediatric nursing and psychiatric experts and included 12 questions about child and parent demographic information, parental access to the Internet, and their ability to use the internet (Marsac et al., 2013; Sveen et al., 2017). The second part was the CRIES-8, which has 8 questions and two subscales; designed for use on children aged 8 years and older who can read independently and includes 4 questions to measure Intrusion subscale and 4 questions to avoidance subscale are hence called CRIES-8. Each question is scored using a 4-point Likert scale of zero (never) to 5 (most of the time) and the total score is obtained by summing the scores of each question. A score above 17 indicates a child with PTSD (Perrin et al., 2005; Verlinden et al., 2014). Children and War Foundation has translated the questionnaire into different languages, including Persian, and its different versions are available on their website.^1^ Also, the Persian version of the questionnaire has been used in several studies and its validity and reliability have been confirmed (Neshat doust et al., 2009; Salari et al., 2017).

In this study to determine the reliability using internal consistency method, the total Cronbach’s alpha correlation coefficient was 0.91, and 0.8 and 0.65 for each of the avoidance and intrusion subscales, respectively.

Also, the Glasgow Coma Scale (GCS) was used to determine the child’s level of consciousness as one of the inclusion criteria. GCS consists of three subscales: eye opening (score 1–4), verbal response (score 1–5), and motor response (score 1–6). Thus, this scale has a range of scores between 3 and 15. The questionnaire’s cut point is 7, and any score below it is deemed to indicate a severe head injury and lowering of consciousness (Hughes et al., 2018; Marcdante et al., 2023).

### 2.5 Procedure

The researcher first prepared a list of eligible patients using patient records and information obtained from nurses in the ward, and after explaining study goals and their willingness to accept or reject participation with written informed consent, the participants were asked to complete a demographic questionnaire by a parent and the CRIES-8 questionnaire by child. After determining the samples based on inclusion criteria, these questionnaires were considered as pre-tests. The participants were assured that their personal information and responses would remain confidential. The control group received no intervention other than the routine care provided by the research setting, in the usual hospital care, the focus of care and education is on the physical aspects of the disease, and in fact, no intervention is done for PTSD. But parents in the intervention group received a four-week child and parent stress training course and psychotherapy online via the researcher’s website.^2^ The educational content of the website was compiled and completed by the researcher and finally designed by a computer engineer under the supervision of the researcher. The website content included topics related to the definition of PTSD, its symptoms in the child, and how to treat the child and help him/her improve mental health. After completing the design of the website, to determine the content validity and final approval, it was reviewed by IT experts, pediatric and psychiatric nursing team members, as well as by an emergency medicine physician and a child psychologist; after that supplementary comments were applied to the structure and content of the site.

The intervention group parents were briefed on PTSD, the need for treatment, and their role in aiding their child during a briefing session. After that, they received the website link and were instructed on how to access the necessary educational materials on different sections of the website. And their questions were answered. The intervention group was notified daily to use the site by the researcher.

The educational content was not limited to written materials, but for a better understanding of the skills suggested on the site, such as mindfulness, videos, and related photos, were also included.

Additionally, upon registration on the site, users were able to communicate with the site manager (researcher) by clicking on the login menu, ask their questions, and receive answers. Table 1 displays the various sections of the website, their contents, and number of questions asked in each section. It should be noted that participants were free to use the website and there was no need to pay. The intervention group was trained for 4 weeks and then for 2 weeks the study samples were given time to influence the intervention. After the sixth week, the level of child stress was again measured by CRIES-8 in two groups and the results were compared. Post-test data were collected by the researcher through telephone, e-mail, and, in some cases, in person.

**Table 1:** Overview of the contents of the website and the number of participants’ questions in each section.

sections	contents	Some of suggestive skills*	Number of questions asked by participants in each section
Recognizing injury and accident	You are not alone Reactions to injury What is the post-traumatic stress reaction? How long does PTSD last? When to worry What happens after the accident?		
Child’s reactions	How is your child?		20
Find a way to help the child get better	What does your child need? How to talk to your child How to deal with new fears and anxieties Work with the child’s healthcare team Injury and pain care When more help is needed self-care	Talking to the child and encouraging her/him to express feelings Encouraging the child and accompanying her/him in writing a story or drawing to help express feelings and cope with stress Mindfulness and its use during exposure	38

*These skills include photos or videos for better learning.

### 2.6 Statistical analysis

Data analysis was done with SPSS version 22 statistical software. Descriptive statistics including mean (Standard Deviation) and frequency (percentage) were used to describe the demographic questionnaire. The normality of quantitative data was checked with Kolmogorov–Smirnov test. Demographic questionnaires of the groups (intervention and control) were compared with Fisher exact test and chi-square test. The overall score of PTSD had a normal distribution, and independent *t*-test was used to compare the groups in terms of the overall score of PTSD and its subdomains. The significance level in all tests was considered *p* < 0.05.

### 2.7 Ethics approval

Ethics have been complied with and approved by the Ethics Committee of Iran University of Medical Sciences (IR.IUMS.FMD.REC1396.9211196239).

## 3 Results

During the study, two participants from the control group and three from the intervention group were excluded from the intervention due to parental withdrawal and lack of cooperation in completing the post-test questionnaire, thus reducing the total number of participants to 105 at the end of the intervention (53 patients in control group and 52 in intervention group). In 56% of parents, mothers were included in the study as the main caregiver of the child. 53.3% of children were female. The mean age in the control and intervention group was 13.15 ± 2.29 and 13.28 ± 2.38, respectively. Most frequent and the three main causes of injuries were car and motor vehicle accidents (32.9%), limb fractures (17%), and falling down (15.4%) in the intervention group, and the car and motorcycle accidents (28.3%), burns (18/95) and limb fractures (17%) in the control group (see Table 2 for more information). In both groups, the majority of parents (>50%) had a high school diploma; the majority of fathers and mothers were employed (89.5%) and housewives (58%), respectively. All parents reported using the website during the intervention (4 weeks). Table 2 shows other demographic characteristics.

**Table 2:** Frequency and percentages of demographic characteristics of intervention and control group.

Variables\groups	Control frequency (percent)	Intervention frequency (percent)	*p*-value
Length of hospitalization (day)			0.221**
3–4	23 (43.4)	18 (34.6)	
5–6	13 (24.5)	21 (40.4)	
7–14	17 (32.1)	13 (25)	
Total	53 (100)	52 (100)	
Economic status			0.358*
Weak	5 (9.5)	4 (7.7)	
Moderate	21 (39.6)	28 (53.8)	
Good	27 (50.9)	20 (38.5)	
Total	53 (100)	52 (100)	
Causes of injuries			0.920*
Falling down	7 (13.2)	8 (15.4)	
Car and motor vehicle accidents	15 (28.3)	17 (32.9)	
Limb fractures	9 (17)	9 (17)	
Sprains	6 (11.3)	5 (9.6)	
Visceral damage	3 (5.7)	5 (9.6)	
Burns	10 (18.9)	7 (13.6)	
Mild head Injuries	3 (5.6)	1 (1.9)	
Total	53 (100)	52 (100)	

*Fisher’s exact test.

** Chi-squired test.

The two groups were similar in all terms of demographic characteristics and based on the results of Chi-squared test, Fisher’s exact test, and independent sample *t*-test there was no statistically significant difference between them (*p* > 0.05).

In the study objectives, as Table 3 shows, based on independent sample t-test, the mean of total stress score and the subscales of intrusion and avoidance were not statistically significant before the intervention (*p* = 0.23, *p* = 0.42, *p* = 0.17 respectively). But after the intervention, the mean score of total PTSD and its subscales decreased in the intervention group and increased in the control group, and the difference between the two groups was statistically significant (*p* < 0.001 for all three variables).

**Table 3:** Comparison of the mean of total PTSD score and its subscales before and after the intervention in control and intervention groups.

Subscales	Time	Intervention group	Control group	*p*-value^b^
		mean ± SD	mean ± SD	
Intrusion	Before education	12.15 ± 3.28	11.64 ± 3.31	0.42
	After education	8.9 ± 3.2	14.33 ± 3.65	<0.001
	*p*-value ^a^	<0.001	<0.001	
Avoidance	Before education	12.76 ± 3.43	11.92 ± 2.93	0.17
	After education	10.42 ± 2.66	14.13 ± 3.41	<0.001
	*p*-value ^a^	<0.001	<0.001	
Total score	Before education	24.92 ± 6.05	23.56 ± 5.56	0.23
	After education	19.32 ± 5.18	28.47 ± 6.51	<0.001
	*p*-value ^a^	<0.001	<0.001	

^a^ Paired *t*-test.

^b^ Independent *t*-test.

Also, Paired sample t-test was used to comparison of means before and after intervention in each group, the results of which showed that in the control group, the mean score of total PTSD and its subscales increased after intervention compared to before; and in the intervention group the mean score of total PTSD and its subscales after the intervention were lower than before the intervention; and differences in both groups was statistically significant (*p* < 0.001).

## 4 Discussion

According to the results of the present study, the most common injuries that children experienced were car or motor vehicle accidents, with approximately 30% of the sample being in car accidents, which is a significant percentage. As the study revealed, the majority of individuals are 10 to 13 years old, and in this age group, the most common accidents that threaten children are accidents with vehicles (Hockenberry et al., 2016). parents neglect to take simple steps that can reduce vehicle injuries to children, such as using a seat belt, not seating children under 13 in the front seat and using a child restraint, as well as failure in the instruction of the traffic signs to help children cross the street and, the lack of strategies and laws that protect children from such injuries can be some possible reasons; as a meta-analysis study showed that almost half of the children with PTSD experienced non-interpersonal accidents, such as a motor vehicle accident (Alisic et al., 2014; Hockenberry et al., 2016). So, training about these causes created by school health nurses for children as well as their parents can be helpful and prevent child injuries.

According to the study, a web-based psychoeducational intervention for parents could have a potential effect on reducing post-traumatic stress symptoms in their children. As in the control group that received routine care in the study environment, their children’s PTSS increased. Therefore, the study’s hypothesis is accepted. Two aspects can be considered when discussing the intervention carried out in this study:

First, the educational intervention was web-based and virtual. In addition to the fact that internet interventions do not require face-to-face referrals, are available, cost-effective and anonymous, they are particularly effective for populations that do not seek treatment other than these training (Kuester et al., 2016). Another feature of these programs that justifies their effectiveness is that it is possible to integrate multimedia components such as photos, videos and audio for better learning. Also, online training allows for standardization of the content of the program, can be tailored to specific needs of age groups and evolutionary stages, as well as social interaction and can also be easily updated. According to the increase of stress symptoms in the children of the control group, as mentioned before there is no intervention or intervention in the hospital to help children’s with PTSD are ineffective.

The results of this study are consistent with several studies demonstrating the importance and usefulness of e-learning for children or parents in the field of PTSD. The study that was most consistent with the present study was research which showed that e-learning for parents of children aged 6–17 with PTSS using the web is feasible, leading to increased parental knowledge and reduced PTSS in children and their parents. However, these effects were only immediately after the intervention and there was no statistically significant difference between the two groups in the follow-up 6 weeks after the intervention (Marsac et al., 2013). The authors of the mentioned study believe that perhaps reducing stress symptoms in children requires more interventions than just parent education alone. In the present study, 2 weeks after the end of the intervention, a significant reduction in PTSS in children was shown, which could be due to a shorter intervention time in the Marsac and colleague’s study (20 min directed use of Educational Website in hospital and 56% use after discharge). Also, in another study, web-based intervention for parents of children with burns and with the aim of reducing PTSD and general stress in parents only a short time after the intervention, immediately after and 3 months after the intervention, was effective in reducing PTSD in the parents; but there was no statistically significant difference between the intervention and control groups in PTSD at 12-month follow-up (Sveen et al., 2017). Therefore, it seems that the control group parents may also seek to increase awareness and receive help to reduce stress symptoms in their children in the long term, but late intervention for stress symptoms can result in devastating consequences. In the present study, 2 weeks after the intervention, the PTSD symptom decreased in the children of the intervention group, but we do not have the follow-up period for the intervention.

Second, the intervention focused on parents, which is the core of child care that is based on family-centered care. The family is the most significant source of support after an injury for children and plays an essential role in their lives. Parents’ anxiety and their inappropriate performance after an injury lead to anxiety and lack of proper adaptation to the injury in children (De Young et al., 2014). If the parents do not recognize the symptoms of PTSD, they do not understand the children’s feelings and do not establish a proper relationship with them. A lack of understanding of the children’s symptoms may lead to blame, or even wrong actions and failure to seek help on time, leading to worsening of symptoms and failure to return to normal. Therefore, if parents are aware of the symptoms of PTSD and are able to provide the necessary support and care for their child, they will be able to improve the child’s symptoms and health. In accordance with the results of this studyweb-based educationalsupport for parents of children with cystic fibrosis have also led to reduced anxiety and depression and improved quality of life in parents (Fidika et al., 2015).

Considering the positive features mentioned above for online education and the importance of educating parents of PTSD children, web-based training can be used as an available and cost-effective method to help children with PTSD. The importance of this intervention doubles if there is no specific program in this field for children and their families during hospitalization.

## 5 Limitations

All parents provided feedback to the researcher that they used the educational site, but there was no strategy for monitoring the intervention group regarding the web surfing period and its use. In addition, sampling was conducted on a convenience and non-random basis; therefore, the generalizations of this study should be taken with caution. Another limitation was the use of self-report questionnaires for assessing PTSS in children.

It is recommended that future long-term follow-up studies be conducted to draw better conclusions about the impact of the intervention. In addition, comparing child and parent training regarding PTSD is recommended. In addition, it would be useful to conduct a study that examines the knowledge and attitudes of pediatric nurses regarding PTSD as well as the measures taken in hospitals to prevent PTSD in children.

## 6 Conclusion

In this study, the findings showed that E-learning parent training has the potential to support children with PTSD. Nurses as members of the health team, play a key role in maintaining and promoting community health by playing roles such as counselor, supporter and trainer in prevention and rehabilitation, they can utilize different educational processes to increase awareness of clients in Promote self-care and use of measures to prevent the spread of disease in the community. Therefore, internet based training is recommended as an easy, accessible and cost-effective way to reduce the symptoms of traumatic stress and possibly increase recovery in such groups of patients. However, it seems that there is still a need for more studies in this field.

## Data availability statement

The raw data supporting the conclusions of this article will be made available by the authors, without undue reservation.

## Ethics statement

The studies involving humans were approved by the Ethics Committee of Iran University of Medical Sciences (IR.IUMS.FMD.REC1396.9211196239). Iran University of Medical Sciences, Tehran, Iran. The studies were conducted in accordance with the local legislation and institutional requirements. Written informed consent for participation in this study was provided by the participants’ legal guardians/next of kin.

## Author contributions

ZO: Conceptualization, Data curation, Methodology, Validation, Visualization, Writing – review & editing. SP: Conceptualization, Funding acquisition, Methodology, Project administration, Resources, Supervision, Writing – review & editing. NS: Investigation, Project administration, Supervision, Writing – review & editing. HH: Formal analysis, Methodology, Software, Writing – review & editing. HN: Data curation, Software, Writing – original draft, Writing – review & editing. All authors read and approved the final manuscript. Also each author agreed both to be personally accountable for the author’s own contributions and to ensure that questions related to the accuracy or integrity of any part of the work, even ones in which the author was not personally involved, are appropriately investigated, resolved, and the resolution documented in the literature.

## Footnotes

^1^ https://www.childrenandwar.org/measures

^2^ www.PTSD-pediatric-nursing-care.ir

## References

[ref1] Alisic E. Zalta A. K. van Wesel F. Larsen S. E. Hafstad G. S. Hassanpour K.. (2014). Rates of post-traumatic stress disorder in trauma-exposed children and adolescents: meta-analysis. Br. J. Psychiatry 204, 335–340. doi: 10.1192/bjp.bp.113.131227, PMID: 24785767

[ref2] Dam R. Robinson H. A. Vince-Cain S. Heaton G. Greenstein A. Sperrin M.. (2019). Engaging parents using web-based feedback on child growth to reduce childhood obesity: a mixed methods study. BMC Public Health 19:300. doi: 10.1186/s12889-019-6618-3, PMID: 30866878 PMC6415344

[ref3] De Young A. C. Hendrikz J. Kenardy J. A. Cobham V. E. Kimble R. M. (2014). Prospective evaluation of parent distress following pediatric burns and identification of risk factors for young child and parent posttraumatic stress disorder. J. Child Adolesc. Psychopharmacol. 24, 9–17. doi: 10.1089/cap.2013.0066, PMID: 24494782

[ref4] Dorrington S. Zavos H. Ball H. McGuffin P. Sumathipala A. Siribaddana S.. (2019). Family functioning, trauma exposure and PTSD: a cross sectional study. J. Affect. Disord. 245, 645–652. doi: 10.1016/j.jad.2018.11.056, PMID: 30445390 PMC6362264

[ref5] Fidika A. Herle M. Lehmann C. Weiss C. Knaevelsrud C. Goldbeck L. (2015). A web-based psychological support program for caregivers of children with cystic fibrosis: a pilot study. Health Qual. Life Outcomes 13:11. doi: 10.1186/s12955-015-0211-y 25652684 PMC4336741

[ref6] Grey M. Whittemore R. Jeon S. Murphy K. Faulkner M. S. Delamater A. (2013). Internet psycho-education programs improve outcomes in youth with type 1 diabetes. Diabetes Care 36, 2475–2482. doi: 10.2337/dc12-2199 23579179 PMC3747907

[ref7] Hockenberry M. J. Rodgers C. C. Wilson D. (2016). Study guide for Wong's essentials of pediatric nursing. Mosby. Missouri

[ref8] Hughes H. K. Kahl L. Harriet Lane Service, (2018). The Harriet lane handbook: a manual for pediatric house officers. Available at:https://books.google.com/books?id=h8SNvgAACAAJ

[ref9] Kaminer D. Seedat S. Stein D. J. (2005). Post-traumatic stress disorder in children. World Psychiatry 4, 121–125. PMID: 16633528 PMC1414752

[ref10] Kassam-Adams N. Marsac M. L. Kohser K. L. Kenardy J. March S. Winston F. K. (2016). Pilot randomized controlled trial of a novel web-based intervention to prevent posttraumatic stress in children following medical events. J. Pediatr. Psychol. 41, 138–148. doi: 10.1093/jpepsy/jsv057, PMID: 26089554 PMC4723670

[ref11] Kenardy J. A. Cox C. M. Brown F. L. (2015). A web-based early intervention can prevent long-term PTS reactions in children with high initial distress following accidental injury. J. Trauma. Stress. 28, 366–369. doi: 10.1002/jts.22025, PMID: 26271018

[ref12] Kovachy B. O'Hara R. Hawkins N. Gershon A. Primeau M. M. Madej J.. (2013). Sleep disturbance in pediatric PTSD: current findings and future directions. J. Clin. Sleep Med. 9, 501–510. doi: 10.5664/jcsm.2678 23674943 PMC3629326

[ref13] Kuester A. Niemeyer H. Knaevelsrud C. (2016). Internet-based interventions for posttraumatic stress: a meta-analysis of randomized controlled trials. Clin. Psychol. Rev. 43, 1–16. doi: 10.1016/j.cpr.2015.11.004, PMID: 26655959

[ref14] Lewis S. J. Arseneault L. Caspi A. Fisher H. L. Matthews T. Moffitt T. E.. (2019). The epidemiology of trauma and post-traumatic stress disorder in a representative cohort of young people in England and Wales. Lancet Psychiatry 6, 247–256. doi: 10.1016/s2215-0366(19)30031-8 30798897 PMC6384243

[ref15] Mahmoudi-Gharaei J. Mohammadi M. Bina M. Yasami M. Fakour Y. Naderi F. (2007). Behavioral group therapy effect on Bam earthquake related PTSD symptoms in children: A randomized clinical trial. Iran J. Pediatr. 16, 1–33.

[ref16] Marcdante K. J. Kliegman R. Schuh A. M. Nelson W. E. (2023). Nelson essentials of pediatrics. Elsevier. Netherlands

[ref17] Marsac M. L. Hildenbrand A. K. Kohser K. L. Winston F. K. Li Y. Kassam-Adams N. (2013). Preventing posttraumatic stress following pediatric injury: a randomized controlled trial of a web-based psycho-educational intervention for parents. J. Pediatr. Psychol. 38, 1101–1111. doi: 10.1093/jpepsy/jst053, PMID: 23912164 PMC3809727

[ref18] Marsac M. L. Kassam-Adams N. Hildenbrand A. K. Nicholls E. Winston F. K. Leff S. S.. (2016). Implementing a trauma-informed approach in pediatric health care networks. JAMA Pediatr. 170, 70–77. doi: 10.1001/jamapediatrics.2015.2206, PMID: 26571032 PMC4939592

[ref19] Moghadam A. H. Sharbaf H. A. M. Mashhadi A. (2017). Effectiveness of eye movement desensitization and reprocessing(EMDR) to reduce the severity of symptoms of post-traumatic stress disorder and stuttering with psychological origin. Arak Med. Univ. J. 19, 87–98.

[ref20] Nakanishi M. Tanaka T. Nishida A. Mandai N. Kitamura N. Yoshii H. (2023). An online intervention to promote mental health literacy for psychosis amongst parents of adolescents: a pilot randomized controlled trial. Early Interv. Psychiatry 17, 737–742. doi: 10.1111/eip.13390, PMID: 36627726

[ref21] Neshat Doust H. Nilforoush Zadeh M. Dehghani F. Molavi H. (2009). Effectiveness of cognitive-behavioral stress management therapy on patients’s quality of life with alopecia areata in skin disease and Leishmaniasis research Centre of Isfahan. J. Arak Univ. Med. Sci. 12, 125–133.

[ref22] Omidvar Ashkalak Z. Nazari H. Seyedfatemi N. Haghani H. Parvizy S. (2020). The effect of parents ‘web-based training on the level of post-traumatic stress symptoms in children: a quasi-experimental study. Available at:https://assets.researchsquare.com/files/rs-17354/v1/1d14d049-ffc1-43ee-af07-9f15cb64e60b.pdf?c=1631831891 10.3389/fpsyg.2024.1325475 PMC11008461 38605831

[ref23] Perrin S. Meiser-Stedman R. Smith P. (2005). The Children's revised impact of event scale (CRIES): validity as a screening instrument for PTSD. Behav. Cogn. Psychother. 33, 487–498. doi: 10.1017/S1352465805002419

[ref24] Salari R. Malekian C. Linck L. Kristiansson R. Sarkadi A. (2017). Screening for PTSD symptoms in unaccompanied refugee minors: a test of the CRIES-8 questionnaire in routine care. Scand. J. Public Health 45, 605–611. doi: 10.1177/1403494817715516, PMID: 28669316

[ref25] Shamsi Pour A. Solgi R. Rozbahani M. Babaee Amirir N. Darabi B. (2019). Effectiveness of of play therapy (with sand play approach) in children with PTSD. J. Except. Child. 18, 55–66.

[ref26] Sugunasingha N. Jones F. W. du Toit G. Jones C. J. (2022). Evaluating an online self-help intervention for parents of children with food allergies. Pediatr. Allergy Immunol. 33:e13731. doi: 10.1111/pai.13731, PMID: 35212055 PMC9306710

[ref27] Sveen J. Andersson G. Buhrman B. Sjöberg F. Willebrand M. (2017). Internet-based information and support program for parents of children with burns: a randomized controlled trial. Burns 43, 583–591. doi: 10.1016/j.burns.2016.08.039, PMID: 28040368

[ref28] U. S. Department of Health and Human Services. (2016). A treatment improvement protocol -trauma-informed care in behavioral health services -tip 57. Available at:https://books.google.com/books?id=4pn4nAAACAAJ

[ref29] Verlinden E. van Meijel E. P. Opmeer B. C. Beer R. de Roos C. Bicanic I. A.. (2014). Characteristics of the Children's revised impact of event scale in a clinically referred Dutch sample. J. Trauma. Stress. 27, 338–344. doi: 10.1002/jts.21910, PMID: 24797017

[ref30] Xiang Y. Cipriani A. Teng T. del Giovane C. Zhang Y. Weisz J.. (2021). Comparative efficacy and acceptability of psychotherapies for post-traumatic stress disorder in children and adolescents: a systematic review and network meta-analysis. Evid. Based Ment. Health 24, 153–160. doi: 10.1136/ebmental-2021-300346, PMID: 34599050 PMC8543231

[ref31] Zhang Y. Zhou X. Yang L. Hetrick S. E. Weisz J. R. Cuijpers P.. (2018). Comparative efficacy and acceptability of psychotherapies for post-traumatic stress disorder in children and adolescents: study protocol for a systematic review and network meta-analysis. BMJ Open 8:e020198. doi: 10.1136/bmjopen-2017-020198, PMID: 29530911 PMC5857664

